# A Case of Primary Melanoma of the Transverse Colon

**DOI:** 10.7759/cureus.1803

**Published:** 2017-10-26

**Authors:** Junaid Raja, Rakesh Hegde, Monica Srodon, Annamika Katoch, Scott Kurtzman, Zhongqiu Zhang

**Affiliations:** 1 Internal Medicine and Radiology, Yale University School of Medicine; 2 General and Colorectal Surgery, Waterbury Hospital; 3 Pathology, Waterbury Hospital; 4 Medical Oncology, Harold Leever Cancer Center

**Keywords:** colorectal melanoma, surgical oncology, non-cutaneous melanoma

## Abstract

Melanoma is among the most prevalent neoplasms diagnosed annually with the vast majority arising from a cutaneous origin. Though there are described metastases to the gastrointestinal tract, there are only rare descriptions of primary gastrointestinal melanoma. Both diagnosis and management of this unique population can be challenging given the infrequency with which it occurs. To follow is the third reported case of transverse colon primary melanoma with a description of multimodality treatment with surgery, chemotherapy, and immunotherapy.

## Introduction

Although cutaneous melanoma is among the top 10 primary malignancies diagnosed annually, melanoma involving the gastrointestinal tract (GIT) is an uncommon problem [1]. Gastrointestinal metastasis of melanoma is quite infrequent, estimates of the incidence of metastasis range from up to 4% in primary surveys to up to 60% in postmortem assessments [2]. Far more unusual still is the incidence of primary mucosal or gastrointestinal melanoma. There have been few case reports of confirmed primary esophageal, stomach, small bowel, and anorectal melanoma in the published literature [3]. The rare descriptions of primary melanoma at these primary sites have been as polypoid, ulcerative, or submucosal lesions and on occasion causing intussusception as a complication [4].

Still given the preponderance of melanoma is of a cutaneous origin it is essential to differentiate gastrointestinal tract melanoma as being primary or metastatic. The evidence for primary disease is strongest in the setting of solitary lesions by endoscopic and/or contrast radiographic findings without evidence of cutaneous disease. Furthermore, given the paucity of evidence of routine pre-melanocytic migration to colonic sites highlights the uniqueness of primary melanoma in the gastrointestinal tract [5].

## Case presentation

A 75-year-old male with a history of hypertension, diabetes, and coronary artery disease presented to the emergency room following a syncopal fall from standing after using the restroom. His history of present illness was remarkable for several weeks of weakness and fatigue coincident with progressive dyspnea on exertion and intermittent dizziness though no concerning features of cardiopulmonary dysfunction or recent infection. Home medications included only metformin and low dose aspirin with no recent changes, a remote smoking history of having quit 20 years prior was noted, no additional substance use, and no personal or family history of malignancy or melanoma. The initial physical exam was unrevealing in examination of all major organ systems including dermatologically no rash, moles, or abnormal lesions were noted, no palpable lymphadenopathy was appreciated, and no physical maneuvers of the abdomen elicited abnormalities or tenderness. Initial assessment for the syncopal episode included a neurologic, pulmonary, and endocrinologic evaluation that did not demonstrate significant abnormalities and a cardiac investigation that was notable for a depressed ejection fraction to 30% and elevated troponins. Of note the patient had an initial anion gap of 22, hemoglobin of 12.8, and a lactate of 4.5. Given the patient also had an elevated TIMI score a heparin drip was initiated and the patient was observed to rule out myocardial ischemia.

**Figure 1:**
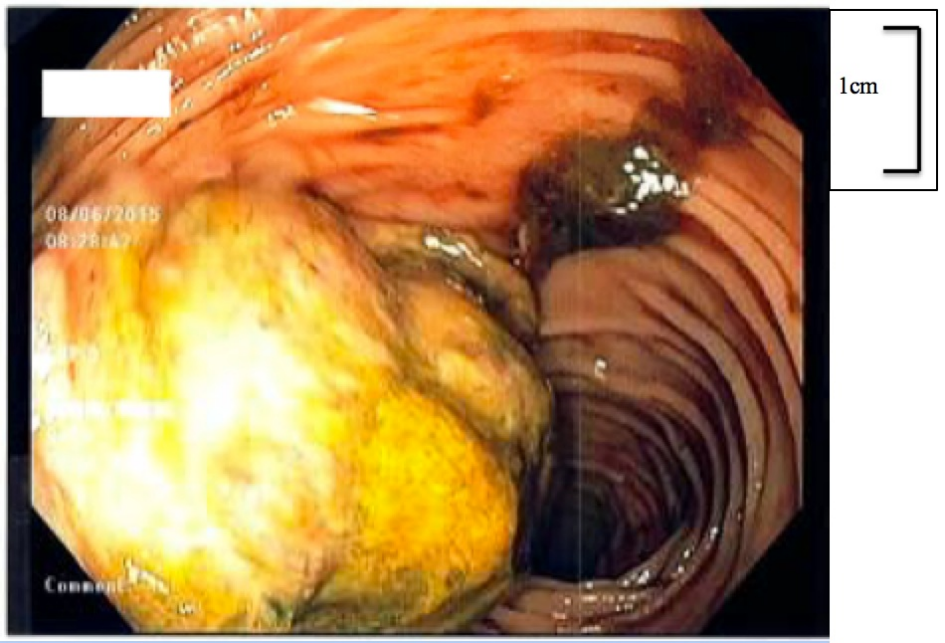
Colonoscopic assessment of transverse colon demonstrating a 7 cm ulcerated, fungating mass occluding the majority of the colonic lumen.

On day two of the admission, the patient experienced two episodes of large volume hematochezia. He denied any prior rectal bleeding or risk factors for upper gastrointestinal bleeding. Esophagogastroduodenoscopy was performed demonstrating a mild hiatal hernia only. Colonoscopy was performed that demonstrated left diverticulosis with bleeding internal hemorrhoids and a 7 cm ulcerated transverse colonic mass occluding the majority of the lumen that was clinically suspicious for malignancy (Figure 1). The lesion was biopsied and tattooed. Pathology revealed a poorly differentiated malignancy which was positive for Human Melanoma Black (HMB-45), Melanoma antigen (Melan-A), and c-kit immunostains and negative for cytokeratin AE/AE3, cytokeratin 7, cytokeratin 20, Cluster of Differentiation (CD34), and Epithelial Specific Antigen (MOC-31), suggesting melanoma. At this time a multidisciplinary team was assembled consisting of hospital medicine, gastroenterology, medical oncology, and surgical oncology to review the case and create a concerted and focused diagnostic and therapeutic plan.

**Figure 2:**
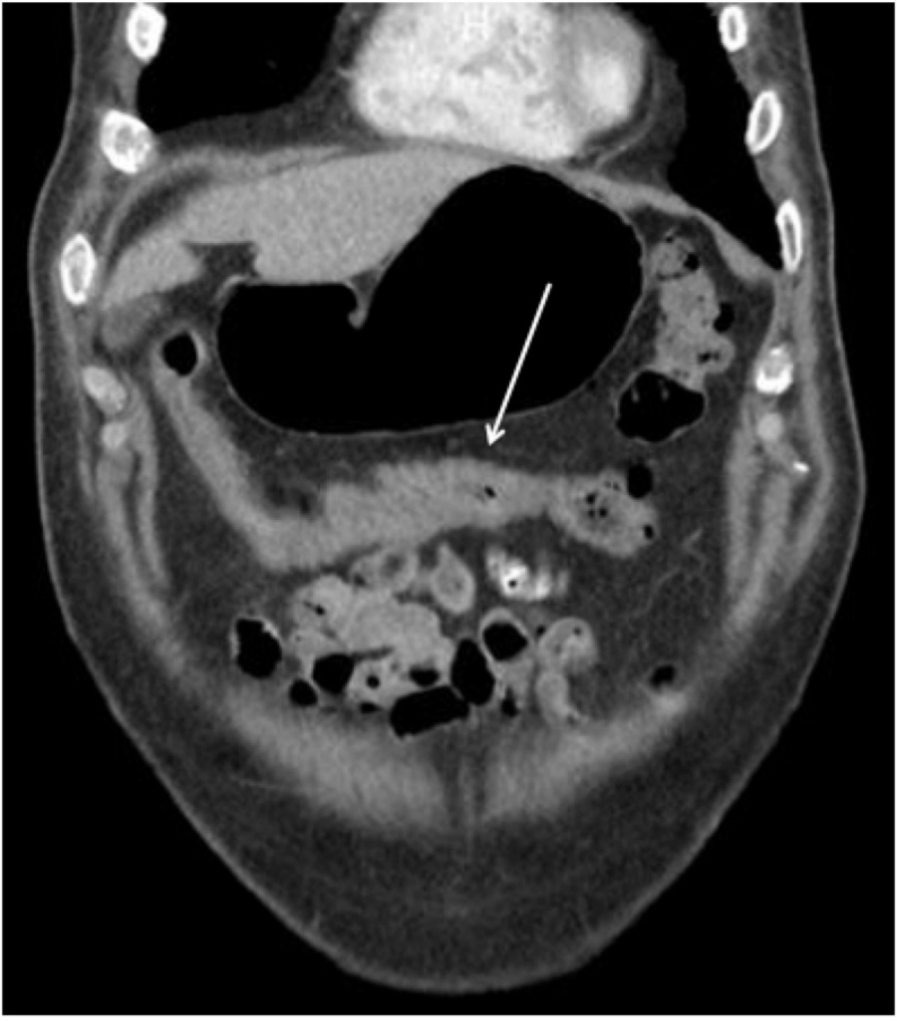
Computed tomography (CT) of abdomen in coronal view demonstrating a large, irregularly shaped lesion of the transverse colon.

**Figure 3:**
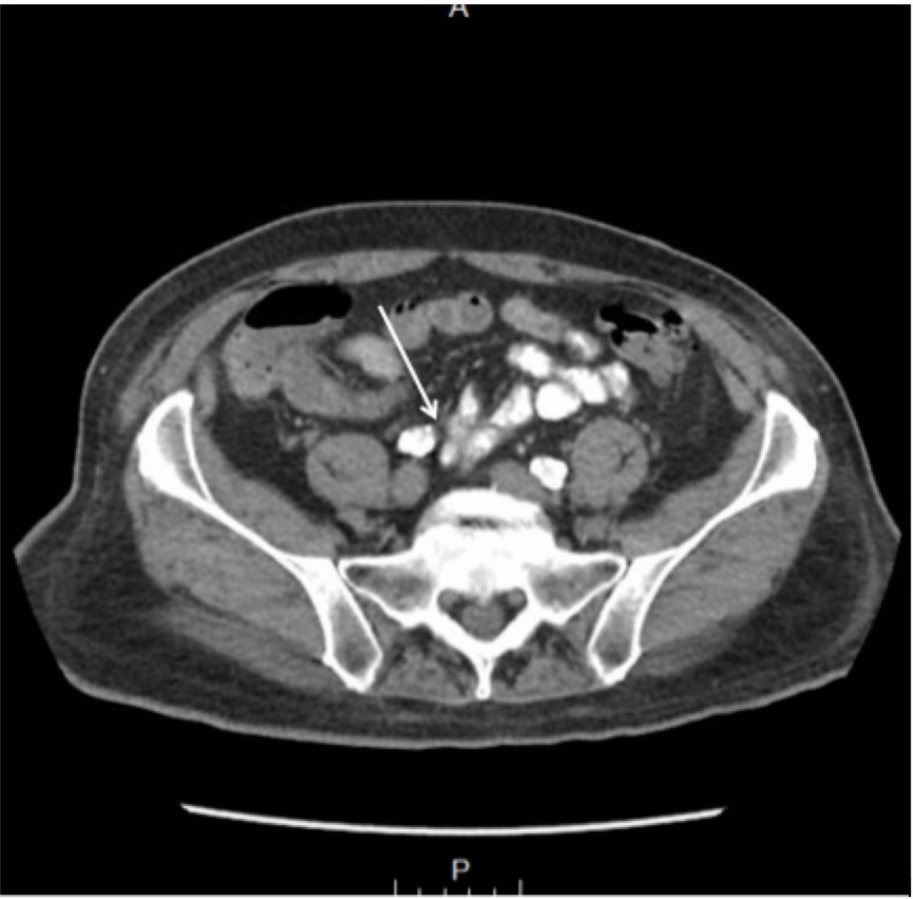
Computed tomography (CT) of abdomen with oral contrast in axial view illustrating filling defect at level of transverse colon.

A computed tomography (CT) scan of the abdomen and pelvis was obtained and demonstrated nonspecific colitis of terminal ascending colon as well as transverse colon but no lymphadenopathy or metastatic sites (Figure 2 and Figure 3). The patient then underwent a laparoscopic ascending right hemicolectomy with an ileocolic anastomosis. The surgical specimen contained a 7 x 5.5 cm tan-red fungating mass with focal hemorrhage that extended 4 cm above mucosal surface.

**Figure 4:**
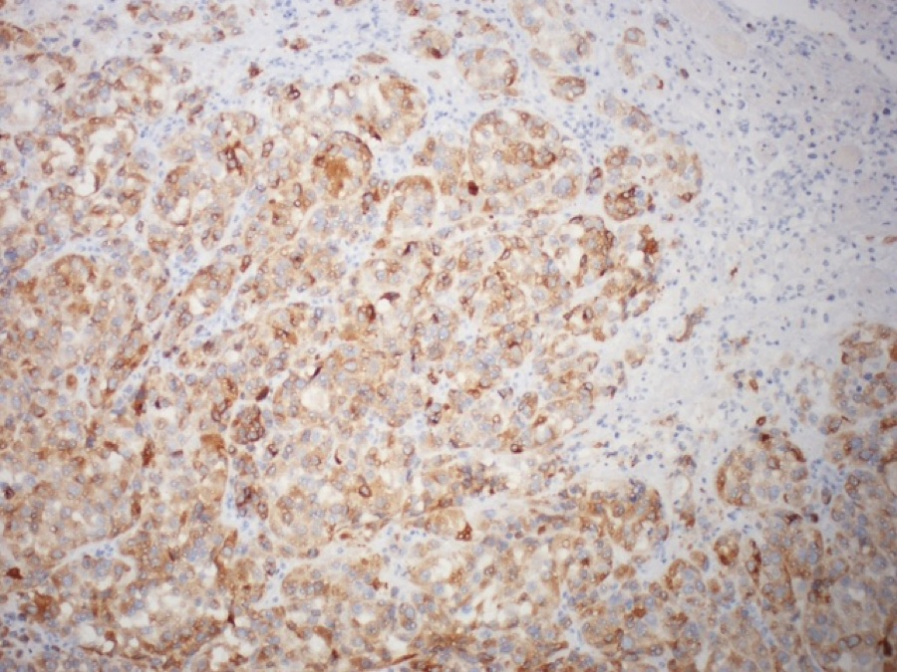
Melan A staining of malignant cells, 40X.

**Figure 5:**
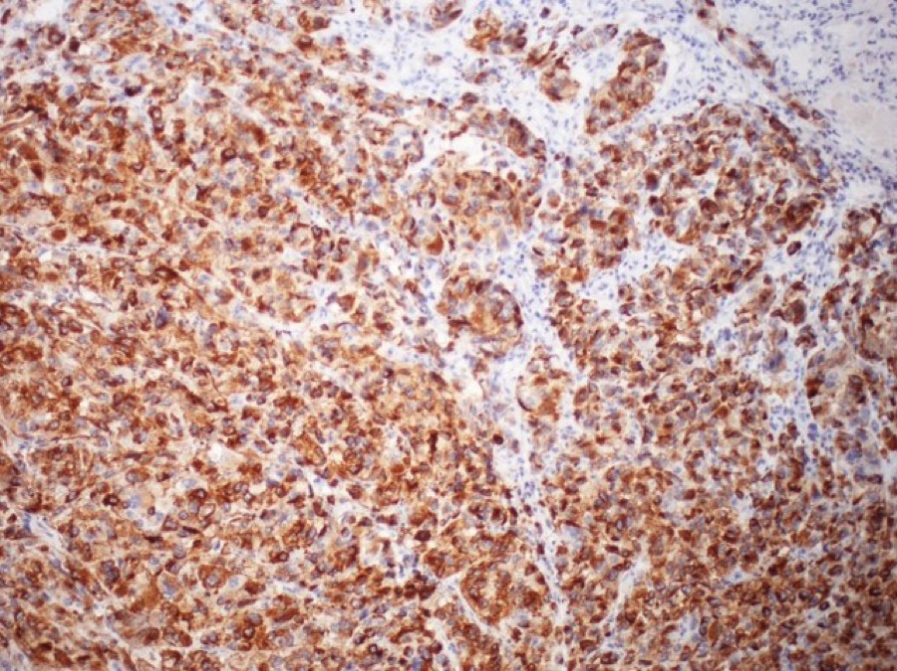
HMB 45 staining of malignant cells, 40X.

The histopathologic analysis of this lesion demonstrated an ulcerated mucosal lesion which invaded through the muscularis. The lesion consisted of a dense infiltrate of cells with large, pleomorphic nuclei with prominent nucleoli. The cells were positive for S100, HMB-45, Melan-A and C-kit by immunohistochemistry and had negative immunoreactions for cytokeratin AE1/AE3, cytokeratin 7, cytokeratin 20, TTF-1, MOC-31, CD34, p63, calretinin, actin and desmin (Figure 4 and Figure 5). The sample was also proto-oncogene B-Raf (BRAF) V600K positive. This constellation of markers was deemed consistent with melanoma. Two out of 15 nodes obtained operatively were also positive for melanoma.

The postoperative course was overall unremarkable and following discharge on postoperative day seven the patient underwent a whole body positron emission tomography (PET)/CT scan that showed only postoperative changes. Ocular and repeat whole body dermatologic evaluation for an alternative primary were negative supporting a diagnosis of primary melanoma of the transverse colon.

## Discussion

Primary colonic melanoma is a rare clinical entity with only 13 cases reported in the published literature. Like any other colonic neoplasm, the clinical presentation of primary colonic melanoma is non-specific. Considering that colonic primary melanoma is an extremely uncommon entity, differentiating primary from secondary melanoma is a challenge. Ozdemir proposed a guideline to differentiate primary bronchial melanoma from secondary melanoma, these same criteria may be applied to colonic melanoma. These are: 1) the lesion must be solitary in the surgical specimen, 2) there must be no previously excised skin melanoma, 3) no previous or concurrent ocular tumor, 4) the morphology must be compatible with that of a primary tumor, 5) there must be no other demonstrable melanoma at the time of surgical exploration, and 6) the findings should be confirmed by careful autopsy for those patients who succumb to disease [6]. Except confirmation by autopsy, all these criteria were met in our patient.

Histopathologic examination of tumor cells shows varying proportions of epithelioid areas and spindle cells. Primary gastrointestinal melanoma is histologically recognized by in situ changes adjacent GI mucosa depicting atypical melanocytic cells in basal layer of epithelium and extending into superficial layers in pagetoid fashion. The tumor cells may show abundant melanin pigment or may be amelanotic. Immunohistochemical stains are highly sensitive for securing diagnosis of melanoma. S-100 is very sensitive in detecting melanoma, HMB-45 and melan-A are highly specific in clinching the diagnosis of melanoma.

Differentiating metastatic from primary GIT melanomas remains a challenge. Primary lesions are much more common in the setting of solitary lesions by endoscopic and/or contrast radiographic findings without evidence of cutaneous disease. Anorectal melanoma is the most common primary gastrointestinal melanoma but accounts for less than 3% of melanomas. It can present with severe abdominal pain, tenesmus, and fatigue. Small intestinal primary melanoma can be associated with intussusception causing small bowel obstruction possibly related to the submucosal location [7].

The proposed mechanism by which bowel mucosa is at risk for melanoma is via migration of neural crest cells from caudal branchial arches as amine precursor uptake decarboxylase cells that can be melanocyte precursors, during embryogenesis [5]. This is why it has been postulated that benign melanosis of the stomach can also occasionally be found [8]. However, there is no established theory or evidence of migration of melanocytic precursor cells to the colon during embryogenesis [5].

The infrequent melanoma metastasis to the colon has been described to appear as polypoid, ulcerative, or submucosal lesions and on rare occurrence cause colonic intussusception [9]. There are only 12 previous cases reported. The addition of a 13th case above (only the third report in the transverse colon) is truly extraordinary [9]. The prognosis of primary gastrointestinal melanoma is poor, although the limited cases of colonic melanoma have had a marginally better outcome compared to other mucosal melanomas that carry a 47% risk of disease-specific mortality [10].

Due to the rarity of this disease, there is no standard treatment guideline proposed for primary colonic melanoma. The few cases in the literature underwent standard partial colectomies of the affected areas. Our patient underwent extended right hemicolectomy with ileocolic anastomosis. Final pathology showed two out of 15 harvested lymph nodes harboring melanoma, with positivity to BRAF. Post-operatively our patient received combination immuno-chemotherapy with sorafenib and dacarbazine.

## Conclusions

Melanoma in the gastrointestinal tract is a rare occurrence in which primary lesions are exceedingly unique. The differentiation between primary and metastatic melanoma of the GIT may be challenging but is essential in formulating a treatment plan. This plan in modern oncology is almost certain to include immunotherapy but the decision regarding additional chemotherapy as well as the role of surgery is significantly impacted by whether the GIT lesion is a primary or metastatic melanoma. As described above, the opportunity to excise a primary melanoma lesion in the GIT prior to metastasis is a rare possibility that may hopefully portend a survival benefit as it did in our patient. However, as a whole, the prognosis for these patients remains poor as many have disease progression at or around the time of diagnosis. As these cases are so uncommon, no standard of care exists but the use of immunotherapy adjuvancy following colorectal surgical resection of the primary mass is both feasible and safe.
